# Advancing African Medicines Agency through Global Health Diplomacy for an Equitable Pan-African Universal Health Coverage: A Scoping Review

**DOI:** 10.3390/ijerph182211758

**Published:** 2021-11-09

**Authors:** Vijay Kumar Chattu, Vishal B. Dave, K. Srikanth Reddy, Bawa Singh, Biniyam Sahiledengle, Demisu Zenbaba Heyi, Cornelius Nattey, Daniel Atlaw, Kioko Jackson, Ziad El-Khatib, Akram Ali Eltom

**Affiliations:** 1 Department of Medicine, Division of Occupational Medicine, Temerty Faculty of Medicine, University of Toronto, Toronto, ON M5G 2C4, Canada; 2 Department of Public Health, Saveetha Medical College, SIMATS, Saveetha University, Chennai 600077, India; 3 Institute of International Relations, The University of the West Indies, St. Augustine Campus, St. Augustine 999183, Trinidad and Tobago; 4 TI Global Limited, 71-75 Shelton Street, Covent Garden, London WC2H 9JQ, UK; optimumshine@outlook.com; 5 Bruyère Research Institute, University of Ottawa, Ottawa, ON K1N 5C8, Canada; skondreddy@bruyere.org; 6 WHO Collaborating Centre for Knowledge Translation and Health Technology Assessment in Health Equity, Ottawa, ON K1N 5C8, Canada; 7 Department of South and Central Asian Studies, School of International Studies, Central University of Punjab, Bathinda 15100, India; bawa.singh@cup.edu.in; 8 Department of Public Health, Madda Walabu University Goba Referral Hospital, Bale-Goba P.O. Box 302, Ethiopia; biniyam.sahiledengle@gmail.com (B.S.); demisu.zenbaba@mwu.edu.et (D.Z.H.); 9 Health Economics and Epidemiology Research Office, Wits Health Consortium, University of the Witwatersrand, Johannesburg 2050, South Africa; cnattey@heroza.org; 10 Department of Anatomy, School of Medicine, Madda Walabu University Goba Referral Hospital, Goba P.O. Box 302, Ethiopia; danielatmwu@gmail.com; 11 Country Medical Services, Ministry of Health, Nairobi 00100, Kenya; kiokojack@yahoo.com; 12 Bill and Joyce Cummings Institute for Global Health, University of Global Health Equity, Kigali P.O. Box 6955, Rwanda; 13 Department of Global Public Health, Karolinska Institutet, 17177 Stockholm, Sweden; 14 Former Federal Minister of Health, Transitional Government of Sudan, Khartoum 825109, Sudan; aeltom@hotmail.com

**Keywords:** African Medicines Agency, health diplomacy, equity, intellectual property rights, universal health coverage, COVID-19

## Abstract

The African continent is home to 15% of the world’s population and suffers from a disease burden of more than 25% globally. In this COVID-19 era, the high burden and mortality are further worsened due to inequities, inequalities such as inadequate health systems, scarce financial and human resources, as well as unavailability of inexpensive medicines of good quality, safety, and efficacy. The Universal Health Coverage ensures that people have access to high-quality essential health services, secure, reliable, and affordable essential medicines and vaccines, as well as financial security. This paper aimed at addressing the critical need for a continental African Medicines Agency (AMA) in addressing the inequities and the role of global health diplomacy in building consensus to support the ratification of the Treaty of AMA. A literature review was done in Scopus, Web of Science, MEDLINE/PubMed, and Google Scholar search engine to identify the critical literature in the context of study objectives. All the articles published after 2015 till 2021 in the context of AMA were included. African Health Strategy 2016–2030 highlighted the importance of an African regulatory mechanism for medicines and medical products. Through global health diplomacy (GHD), the African Union and its partners can negotiate and cooperate in providing infrastructural, administrative, and regulatory support for establishing the AMA. The paper emphasizes the South–South cooperation and highlights the contributions of India and China in the supply of medicines and vaccines to Africa. A strong AMA created through GHD can be a vital instrument in utilizing Trade-Related Aspects of Intellectual Property Rights (TRIPS) flexibilities extension and an ideal partner for European and other regional regulatory authorities seeking to stem the tide of counterfeit, sub-standard, or fake products.

## 1. Introduction

The African continent is home to 15% of the world’s population and suffers from a disease burden of more than 25% globally [1,2]. The continent faces the triple burden of infectious diseases, non-communicable diseases (NCDs), and nutritional disorders [3]. Africa’s high disease burden and high mortality due to preventable and curable diseases are partly due to inadequate health systems, scarce financial and human resources, as well as unavailability and inexpensive medicines of good quality, safety, and efficacy [4]. Furthermore, the COVID-19 pandemic has exacerbated the continent’s health disparities resulting from inequalities and injustice, impeding regional development. For many years, sub-Saharan African countries’ capacity to regulate medicines has been hampered by fragmented legal systems, ineffective management systems, processes, and shortage of personnel and resources, resulting in the subsequent proliferation of sub-standard and fake medicines in the markets [5,6]. According to WHO, universal health coverage (UHC) aims to ensure that everyone has access to the health care they need without facing financial hardship. Therefore, UHC ensures that people have access to high-quality essential health services, secure, reliable, and affordable essential medicines and vaccines, as well as financial security [7]. The African Health Strategy (AHS) 2016–2030 highlighted the importance of an African regulatory mechanism for medicines and medical products. During the 32nd Assembly of the African Union, the African Medicines Agency (AMA) was founded in 2019. It clearly stated that UHC “must be supported with an adequate supply of commodities including essential medicines, contraceptives, condoms, vaccines and effective drugs and other supplies” [8]. The AHS 2016–2030 further emphasized that the “regulation of medical products and technologies at the continental level should be prioritized to support the availability of quality products.” Endorsed by the African Ministers of Health in May 2016, the AHS 2016–2030 further identifies “Regulation of medicines and health products” among the areas which could benefit most from inter-country collaboration within the continent [8]. Moreover, in this post-COVID-19 era, with the geopolitical shifts and the changes in the global order, there is a great need for regional strengthening to address the systemic post- pandemic challenges such as socio-economic inequities, gender inequalities, regionalism, racism, and injustice towards vulnerable groups. Therefore, this paper attempts to address the main research question, “Can the inequities be addressed through the continental AMA, and if so, how does the AMA aid to achieve an equitable pan-African UHC?” In this context, the main objectives of this review are (1) to reaffirm the critical need for a continental AMA for aiding equity, (2) to underscore the role of global health diplomacy (GHD) supporting this new governance structure towards ratifying the Treaty of AMA, and (3) to explore how AMA can benefit from the regional South–South cooperation and other global partnerships to address its challenges through GHD during this pandemic.

## 2. Materials and Methods

A scoping review approach was employed to answer the research question using the available published evidence and various statistical reports. A scoping review is a relatively new approach to evidence synthesis and differs from systematic reviews in its purpose and aims [9]. It provides an overview of the available research without producing a summary answer to a discrete research question [10]. Since the emergence of AMA has been a developing concept for the past few years, the scoping review seems to be an appropriate methodology to summarize and give a snapshot based on the available published literature from relevant sources. Therefore, we employ this technique to provide critical insight into this critical study during this COVID-19 pandemic. Additionally, the scoping reviews describe the existing literature and other sources of information commonly include findings from studies with a range of designs and methods [11]. This scoping review can be particularly useful when the information on a topic has not been comprehensively reviewed or is complex and diverse [12] as in our current topic of the African Medicines Agency. This method also enables the research topic to be expanded in time and across geographic boundaries to improve existing understanding. The well-debated topic of AMA and its advantages for the African region becomes the right topic for conducting cross-disciplinary perspectives among the domains of politics, policymaking, public health, ethics, management, and others. We, therefore, have conducted an extensive literature search in all popular databases for this scoping review which investigated relevant articles and reports that recommend the need for a continental governance mechanism through a pan-African regulatory agency of medicines and drugs in Africa. The advantages of this type of literature review are: (1) it requires more comprehensive and structured searches of the available literature to maximize the capture of appropriate information, (2) it provides reproducible results, and (3) it decreases the potential bias that may result from flawed implementations [13]. Therefore, this review provides an updated account of available information, enables cross-disciplinary research, and shapes new research avenues on the evolution and the need for AMA.

### 2.1. Literature Search

We followed Arksey and O’Malley’s framework for scoping reviews [14] and the PRISMA-ScR (Preferred Reporting Items for Systematic reviews and Meta-Analyses extension for Scoping Reviews) checklist in reporting findings [15]. During this pandemic phase, it is very critical and beneficial to provide the summary of all the perspectives of global stakeholders, the regional bodies, and national governments as the pandemic-related events are unfolding. The reviewing framework consisted of the following steps: (1) Identifying the research question, (2) Identifying relevant studies, (3) Study selection, (4) Charting the data, (5) Collating, summarizing and reporting the findings. The articles of potential interest were identified by searching the popular databases such as Scopus, Web of Sciences, PubMed, MEDLINE, and Google Scholar search engine using the following search strategy using the Boolean operators. Several keywords were used such as (Africa Medicines Agency AND Regulatory Harmonization AND (Regional Regulatory Agencies OR National Regulatory Agencies) AND (Medical Products Regulations) AND (Health Governance OR Health Diplomacy) AND Africa*. The search was then refined by identifying articles that discussed the role of regional regulatory agencies, regional health governance, the need for regulatory harmonization, the Africa Union’s Treaty to AMA, and pharmaceuticals regulations.

### 2.2. Inclusion Criteria

Only articles written in English were considered, and all the articles published between January 2015 and 30 May 2021 in the context of the proposal and evolution of the AMA were included. Though the emphasis was given to published articles, some authentic websites of the Africa Union Commission, Ministries of Health, and WHO, as well as other annual reports of AU were also included.

### 2.3. Selection of Studies

To ensure the quality of the articles included in the study, two researchers (VKC and KSR) independently assessed them by screening the titles and abstracts. They also excluded the duplicates of the articles or reports from the final search. A total of 74 publications (articles, reports, and websites) were included in this review, and those non-relevant to the African context were excluded. This review provides deep insight into the ongoing process of AMA ratification and GHD negotiations while drawing parallels with the previous regional agreements and current publications. The paper explores the role of AMA and the critical need for it during this pandemic phase through the standpoints of equity and bioethics, by exploring contemporary research in a dynamic manner.

### 2.4. Quality Assessment

The methodological quality of each study was assessed using the PRISMA-ScR checklist in reporting findings. The included studies’ quality was also assessed using the Strengthening the Reporting of Observational Studies in Epidemiology (STROBE) scale.

## 3. Results

**Figure 1:**
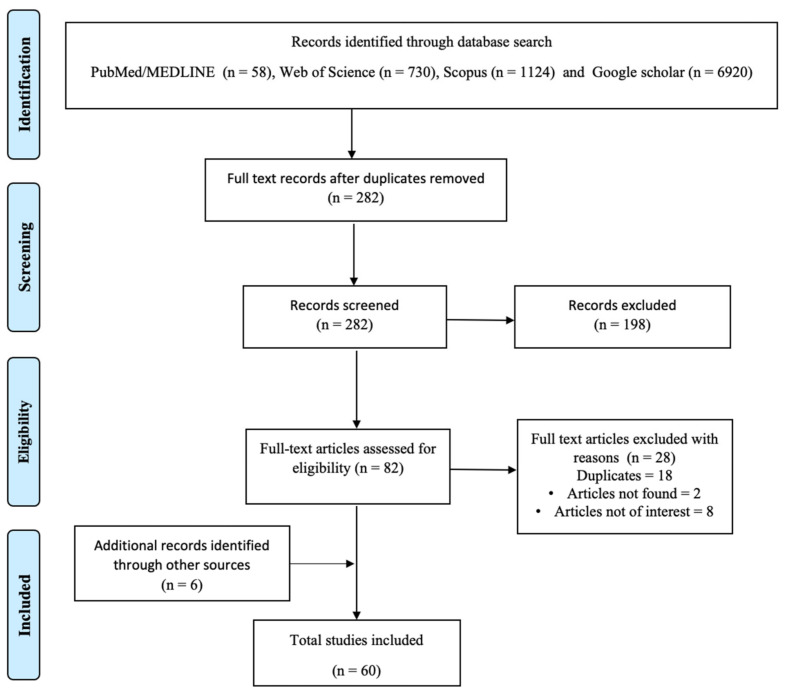
PRISMA flow diagram for the scoping review process.

Of the total 8832 studies that were obtained through database searching, after excluding irrelevant titles and duplicate records, a total of 282 abstracts were considered for screening. Of these, 82 studies were considered for the full-text review, and 28 were excluded for various reasons. There were additional 6 records identified through additional sources. Finally, a total of 60 articles were considered for this scoping review (Figure 1).

The main findings from the search were classified into various subsections and discussed below. They are broadly classified into (i) Regulation of Medical products, the current regulatory mechanisms, (ii) the evolution of AMA, (iii) the growing importance of GHD, relevant to the functions of the proposed AMA and its governance, (iv) anticipated benefits of fully functional AMA.

### 3.1. Regulation of Medical Products in Africa

According to a study published in 2010, 7% of the 46 countries in sub-Saharan Africa have moderately established medicine regulatory capability, about 63% have limited capacities, and the remaining 30% have a National Medical Regulatory Agency (NMRA) in place [16]. Building up the medical products regulatory capacity is vital to the achievement of the UHC, the Sustainable Development Goals (SDGs) 1 and 3, including people’s access to quality, safe, and effective health products and the AU’s Agenda 2063 aspirations [17]. Restricted regulatory ability is related to the circulation of medical goods that do not meet quality, safety, and efficacy resulting in negative public health and economic consequences. A recent exploratory study by Sigonda et al. assessed the effect of the East African Community medicines regulatory harmonization initiative on the capacity of six NRMAs found that the initiative has contributed to improved capacity to regulate medical products [18].

### Regulatory Harmonization Process in Africa

The African Medicines Regulatory Harmonization (AMRH) Initiative is an effort by the African Union to improve regulatory capability, promote regulatory harmonization, and speed up access to high-quality, safe, and effective medicines [19]. The initiative is part of the African Union’s Pharmaceutical Manufacturing Plan for Africa (PMPA), a policy mechanism that seeks to create an encouraging regulatory climate for local production while contributing to the UHC, AU Agenda 2063, and SDGs targets [20,21]. The AMRH initiative is also being carried out in some of Africa’s regional economic communities (RECs), such as the Economic Community of West African States, the Intergovernmental Authority on Development, and the Economic Community of Central African States. More than 85% of sub-Saharan African countries are covered by it, with varying degrees of implementation. The AMRH Initiative developed a Model Law on Medical Products Regulation to resolve incoherent medicines laws in African countries, ensuring successful regulation and harmonization [22,23]. The AMRH serves as a forum for RECs, Regional Health Organizations (RHOs), and the AU Member States to harmonize medicine regulation and develop and improve regulatory capacity. These aims are accomplished by enlisting interested governments, sponsors, and other stakeholders to invest in regulations of drugs and medicines [21,24].

### 3.2. Evolution of African Medical Agency (AMA)

**Figure 2:**
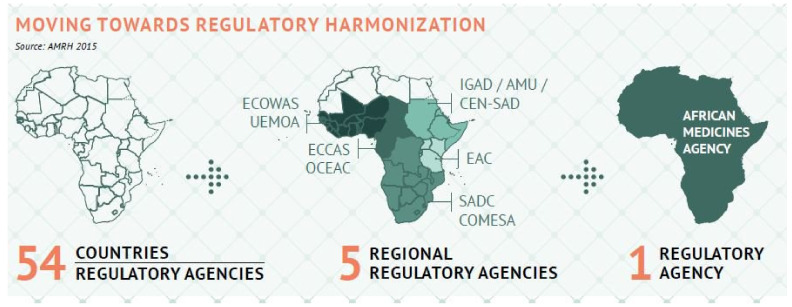
Evolution of African Medicines Agency. Source: AMRH, 2015.

The AMRH Initiative also lays the groundwork for the AMA (Figure 2), created by AU Executive Council Decision EX.CL/872(XXVI) in January 2015 [22,25]. These measures are expected to speed up research and development of new or enhanced medical therapies for poverty-related neglected diseases, create an enabling climate for local manufacturing, and contribute to the AU Agenda 2063, as well as the global 2030 SDGs [21,22]. The AMA was founded two years ago at the AU’s 32nd Assembly held in Addis Ababa on 11th February 2019. It was hailed as a key tool for enhancing regulatory oversight of medicines and vaccines across the continent’s 54 countries [26]. The COVID-19 pandemic highlighted the need for a regulatory body such as the AMA to fill gaps and inconsistencies in the current patchwork of regulations between the continent’s five regional regulatory authorities and dozens of national authorities [27].

### 3.3. The Growing Importance of Global Health Diplomacy in Africa

**Figure 3:**
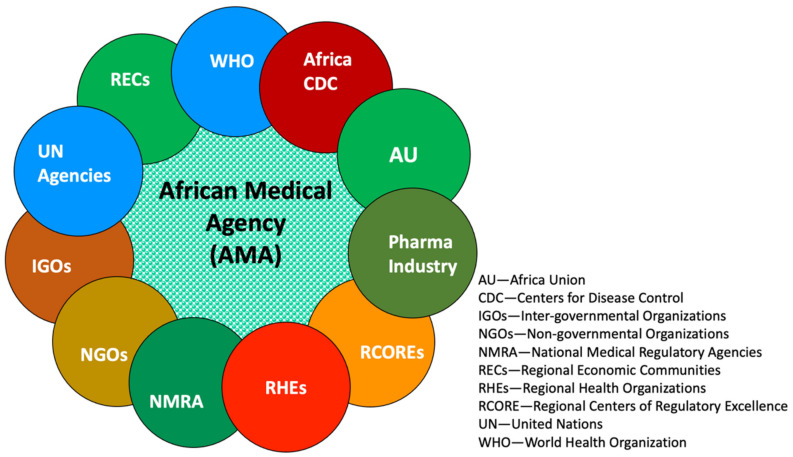
Engagement of African Medical Agency in health diplomacy with its main regional partners. Source: conceptualized by the authors.

**Figure 4:**
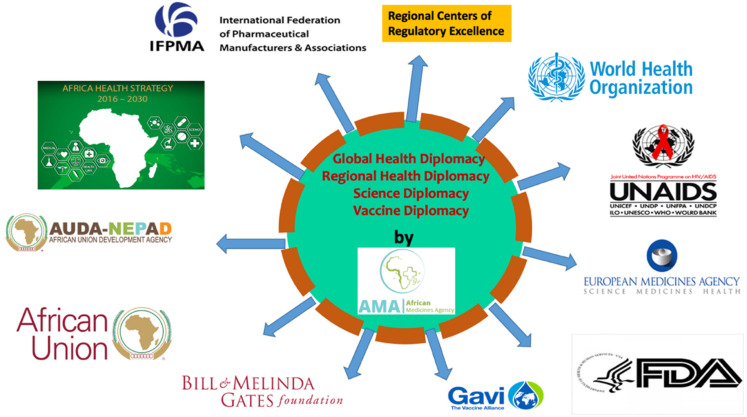
Engagement of African Medicines Agency in regional health/global health diplomacy with international partners. Source: conceptualized by the authors.

GHD refers to “multi-level, multi-actor negotiation processes that shape and manage the global policy environment for health.” GHD’s core principle is bringing nations together in the diplomatic fora to tackle public health problems, including establishing new organizations and initiatives [28]. The COVID-19 pandemic, while reaffirming the role of GHD for a global response, also necessitated the regional health diplomacy for the regional responses, such as the EU vaccine initiative independent of the WHO and the AMA. However, in realizing a fully functional Agency, health diplomacy at multiple levels, particularly the regional level within the AU constitutional framework (Figure 3), is vital [29]. Article 3 of the Constitutive Act of AU’s objective is to work with relevant international partners to eradicate preventable diseases (Figure 4) and promote good health on the continent [30].

#### Establishment of African Medicines Agency

The Treaty for establishing the African Medicines Agency is an international treaty, pending ratification and accession by at least 15 member states of the AU, to establish the AMA as its specialized agency. As of 20 March 2021, only eight countries have so far ratified the AMA Treaty. While the remaining seven countries consider ratifying the Treaty, the African member states also need to negotiate a legal framework and resolve the conflict of interests related to funding. Bilateral and regional health diplomacy is a way forward for consensus building among African member states moving forward.

Both bilateral diplomacy and regional health diplomacy were found to build new partnerships and initiatives for health earlier across the regions, including Africa. Bilateral health diplomacy has helped establish bilateral health partnerships to address various health issues, including disease outbreaks. For example, the US and China developed a health partnership in response to SARS. However, the US and China’s bilateral health diplomacy response to SARS was driven more strategically than health interests [29]. At the same time, while engaging in existing regional social policies, regional health diplomacy contributes to the evolution of new health policies that promote access to healthcare and medicines in the region [31]. In Africa, regional health diplomacy has helped establish a US 200 million new health financing initiative to achieve UHC across Africa’s 54 countries [32]. The high cost of drugs and medical supplies is one of the significant challenges of small African island states. To overcome this challenge, seven small African island states such as Cabo Verde, Comoros, Madagascar, Mauritius, São Tomé and Príncipe, and Seychelles signed the Pooled Procurement agreement in 2020, which stands as the testament for regional health diplomacy in the region [33].

The G7 and G20, which played a pivotal role in establishing global institutions and initiatives such as the Global Fund, could serve as venues for multilateral negotiations and cooperation in establishing the AMA that harmonizes medical regulation in the continent and serves the African population. African regionalism is the driving force for Africa’s global health diplomacy [34]. The regional vision for a healthier population would propel the bilateral and regional negotiations with state and non-state actors to establish AMA.

**Figure 5:**
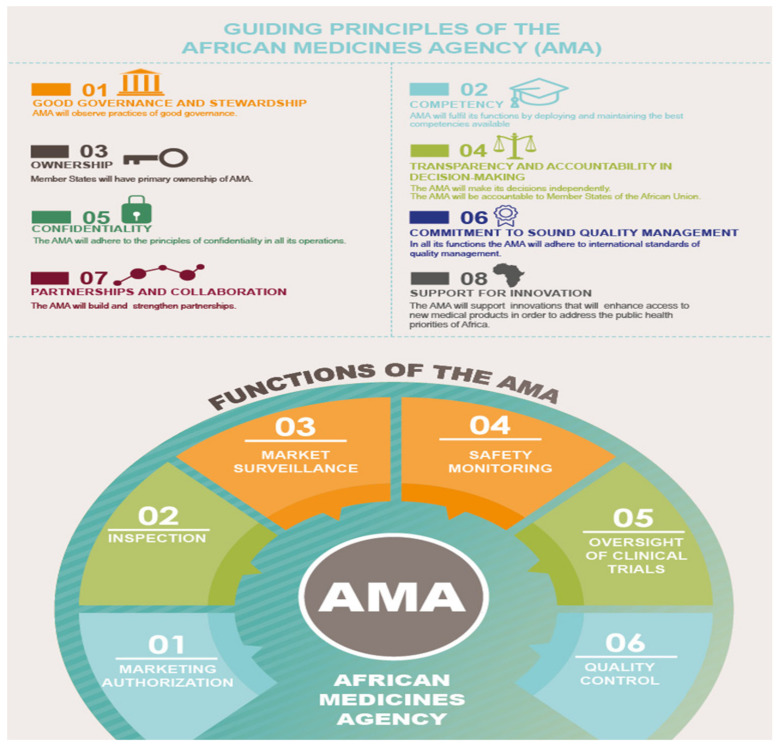
Guiding principles of African Medicines Agency. Source: AUDA-NEPAD https://nepad.org/microsite/african-medicines-agency-ama (accessed on 22 May 2021).

According to Article 6 of the Treaty of the AMA, 18 functions were listed in the Treaty [35]. The summary of AMA’s main functions (Table 1) and guiding principles (Figure 5) are shown below.

### 3.4. Anticipated Benefits and Advantages of Fully Functional AMA

#### 3.4.1. Growth of Local Pharmaceutical Manufacturing Capacity

One of the most important PMPA’s objectives is to increase the continental manufacturing capacity. PMPA acknowledges the global lack of faith in Africa’s regulatory capacity. Therefore, robust AMA can underpin pharmaceutical manufacturing growth [36]. Being a regional agency, AMA can also be in a prime position to support the capacity development work of the South African Generic Medicines Association (SAGMA), which delivers Good Manufacturing Practices (GMP), the WHO Pre-Qualification, key policy briefings of intellectual property rights (IPR), and Trade-Related Aspects of Intellectual Property Rights (TRIPS) flexibilities to manufacturers across the South African Development Community (SADC) region. On the other hand, intending to learn from common challenges, three regional pharmaceutical manufacturers associations came together to form the Federation of African Pharmaceutical Manufacturers Associations (FAPMA). Once again, AMA holds the great opportunity to support these capacity-building initiatives by inducing robust regulatory mechanisms. Stronger regulatory frameworks also encourage international monetary confidence and, therefore, a surge in the manufacturing capacity.

#### 3.4.2. Promoting Regulatory Cooperation and Harmonization

a. Adopting AMRH’s workstreams, AMA would facilitate the medicines evaluation and registration through Common Technical Document formation. Currently, the marketing authorization can vary from 4 to 7 years in line with the resourcefulness of the individual NMRA. This lengthy period has adversely impacted the pharmaceutical companies in supplying medicines to the continent [37]. Mary Ampomah, President and CEO of the Global Alliance of Sickle Cell Disease Organizations (GASCDO) stressed the value of getting access to safe drugs and a chance for a better life in Africa. She highlighted that the AMA would speed up the approval process, and as a result, the medicines will be available sooner to the people.
b. Although adopted in February 2019 by the member states of AU, as of February 2021, only eight members out of the required fifteen member states have ratified; a required legal process before AMA could come into effect. One of the challenging factors is the lack of legislation and how it would affect the activity and authority of NMRA in any given member state. However, learning from Ghana’s experience in harmonizing their legislation to AMA, member states must provide the required coordination and recognition of common purpose from the Ministry of Health (parent ministry), current Government, Ministry of Finance, Ministry of Foreign Affairs, and the Office of Attorney General [38]. Despite being a complex process, this now forms the legitimate platform effort to adopt and utilize AMA’s objectives to benefit Ghana’s population. This is paramount as there is a major variation among what is being regulated by NMRAs across Africa. As noted by Ndomondo-Sigonda et al., out of 26 NMRAs, only 15% are mandated to perform every regulatory activity, while 65% control veterinary medicines, 69% regulate herbal medicines, and more than two-thirds regulate items that include foods, pesticides, poisons, bottled water, cosmetics, and animal food supplements [37]. Therefore, harmonization through a common regulatory framework can prevent further delays in bringing important medicines to the public.
c. Other regulatory functions of pharmacovigilance (PV) also showed a significant gap as only eight countries out of 26 sub-Saharan African countries had collected data on adverse events. Varying proportions of countries showed poor post-marketing surveillance (PMS), leading to SSFFCs (substandard, spurious, falsely labeled, falsified, and counterfeit) medicines being available in the market. AMA is expected to ease the process by bringing different technology strengths, technical expertise, and shared human and financial resources.
d. Impact on training and education: The establishment of RCOREs meets training and education standards as these RCOREs possess the expertise on one or more aspects of the capacity building for NMRAs, i.e., training, education, regulatory, manufacturing, quality assurance, and control.

#### 3.4.3. Continental Relevance of Priorities

We can gain a continent-wide perspective by evaluating the medical products of diseases that most affect Africa’s population. As determined by the AU, the resources are directed to understand, evaluate, and authorize medicines and medical products, elevating people from ill health on the continent. Neglected-disease products have seen the alternative regulatory arrangements through EMA Article 58, the WHO Prequalification Scheme, and the United States Food and Drug Administration Tentative Approval [37]. Dr. Yaw Asare-Aboagye, Head of Regional Clinical Operations at the Drugs for Neglected Tropical Diseases Initiative, believes that AMA can speed up medicine delivery, provide clear guidelines, and encourage information to be shared for the benefit of science.

#### 3.4.4. Information Availability

One of the biggest barriers to understanding and enabling the effective solution has been the lack of information, especially with timely access. This limitation creates fertile ground for illicit traders and beneficiaries when coupled with fragile national regulatory systems (NMRA) and porous borders. The two recent pandemics, Ebola and COVID-19, have reinforced the significance of timely access to accurate and adequate information. Therefore, AMA will inspect, coordinate, and share vital information in propelling the usage of the most appropriate and effective medicines and health products.

#### 3.4.5. Ensuring Robust Response to Counterfeit Medicine

AMA will coordinate prevention, detection, and response to counterfeit and falsified medicines. As highlighted in Appendix A, illegitimate operators are taking advantage of the present pandemic. Within one year, about 28 countries have reported news on diverted and/or substandard and falsified vaccines in the lay press, many available online. On 3 March 2021, law enforcement agencies raided two warehouses and seized 400 vials 2400 falsified COVID-19 vaccine doses [39]. This raid demonstrated the nature of the cross-border activity as the South African teams were supported and facilitated by Interpol. Under the already delicate environment of safety, the public can lose trust with unfavorable outcomes, which in return could damage the vaccine roll-out. AMA could serve as the continental focal point, coordinating the alerts and interventions with other world regions. Therefore, AMA can effectively restrict the introduction, circulation, or growth of SSFFC (substandard, spurious, falsely labeled, falsified, and counterfeit) medical products by receiving and disseminating information from international partner NMRAs within Africa.

## 4. Discussion

As the AMA is modeled after the European Medicines Agency (EMA), health diplomacy would also help the AU and the European Union negotiate and cooperate in providing infrastructural, technological, administrative, and regulatory support to establish AMA in Africa. AMA will play a key role in combatting the continent’s widespread use of sub-standard and falsified medical goods, which has worsened after COVID-19. Members of the African Union have largely seen the geographical borders remain opened and supportive of trade, business, and migration. While the economic and social cohesion has increased, the continent requires the mechanisms to ensure the necessary quality and timely access to essential items like medicines, vaccines, and medical products. AMA would underpin the sustainability and enhancement of the continental development that has already taken place in the past two decades.

### 4.1. Enhancing the Role of a Clinical Trial in Shaping the Population Health

Continental coordination of clinical trial applications can benefit from the joint panel reviews. This process is expected to share and utilize continental expertise and efficient use of financial and human resources.

### 4.2. Greater Coordination among African Countries

United to ensure timely access to safe, quality, and effective medicines and medical products, the AMA as a vehicle optimizes the synergy of coordination. This not only reduces the burden on NMRAs but also ensures the effectiveness of various interventions. Within seven years, the world is going through a second Public Health Emergency of International Concern (PHEIC). While, as a world, we are still in the process of implementing concerning interventions for the first PHEIC, Ebola crisis, the second PHEIC, COVID-19 pandemic is already costing millions of lives with potentially long-term damage to human developments. According to the IFPMA and IAPO webinar on AMA on 10 December 2020, the representative of NEPAD reiterates AMA’s importance as NMRAs face a lack of technical, financial, and human resource capacity. One example given was the lack of ability to assess the in vitro diagnostic test for COVID-19 by individual countries.

On the other hand, as the Medical Product Quality Report identified, medical products undergo price hikes, especially when the demand exceeds the supply. Therefore, as a regional regulatory authority, AMA, in collaboration with NMRAs, can better inform pricing, logistics, and distribution. As highlighted by Singh et al., the sub-Saharan African countries were ignored during the COVID-19 vaccine roll-out, and the COVID-19 Supply Chain Taskforce had facilitated a shipment of WHO medical cargo from Dubai to Addis Ababa by mid-April 2021, and the medical supplies were transported to several parts of Africa [40]. In this context, Molinaro has argued that if these timely intervention measures had not taken place, about 300,000 to 3.3 million Africans would have died [41].

### 4.3. Efficient Implementation of Intellectual Property Rights

The TRIPS agreement is a central mechanism in facilitating the global trade from particulars concerning their IPRs. These multilateral legal agreements are designed to protect IPRs while balancing to meet vulnerable countries’ needs and make the medicines as accessible as possible. Doha Declaration of TRIPS agreement and Public Health has been the historical achievement in influencing equity in access to medicines. However, applying the TRIPS flexibilities requires a member state to implement these into their domestic law system [42]. Therefore, as M. Monirul Azam noted, the country must possess adequate regulatory and institutional capacities to take advantage of TRIPS flexibilities [43]. Although for the least developed countries (LDCs), the 2001 Doha Declaration facilitated affordable medicines until January 2016, it lacked the adoption. As a result, AU and NEPAD expressed their concerns about the lack of adoption of TRIPS flexibilities into the domestic law due to weaker political willingness and/or lack of knowledge among technocrats along with fragile legal and regulatory capabilities [36].

Furthermore, under the provision of TRIPS flexibilities, some LDCs do not constitute a large enough population and, therefore, fall short in harnessing the economies of scale for purchasing power and local production. Under these requirements, AMA with strong regulatory expertise and harmonized legal frameworks would enhance successfully adopting TRIPS flexibilities. For the health benefit of the African population, AMA can be a vital instrument in utilizing TRIPS flexibilities extension, which now runs till 1 January 2033 [36]. This extension’s central objective necessitates the capacity building of manufacturing, regional regulatory capacity, and judicial IP law interventions in the LDCs. In this context, a recent article by Chattu et al. argues that the IP regime should not become a barrier to the availability and affordability of COVID-19 medical equipment and vaccinations and urged that the countries that rejected the joint proposal of TRIPS waiver by India and South Africa should reconsider and lend their full support at the World Trade Organization (WHO) [44].

### 4.4. South–South Cooperation and African Medical Agency

The pandemic has not only changed the world order but also reset the bilateral, regional, and multilateral engagements, which have also been undergoing a tectonic shift. India and China have been proactively pursuing health diplomacy towards Africa [45,46,47]. Therefore, in this section we discuss the new strategies for effective functioning of AMA through its partnerships with various regional and global players. A special emphasis is laid on the South–South cooperation (among global south) and therefore, we would examine (1) the key features of health diplomacy by India and China towards Africa and (2) the impacts, challenges, and opportunities that are likely to emerge from the existence of AMA through this health cooperation. Tediosi et al. have explored the BRICS’ engagement in the global movement for UHC and the implications for global health governance. Their study found that though BRICS are unlikely to be a unified political block that will transform global health governance, individually they are influential players in global health by giving greater voice to the LMICs [48].

### 4.5. Impact of India’s Health Diplomacy

Against the background of COVID-19, India has been pursuing its health diplomacy towards the African continent. At the peak of a pandemic during the first wave, when other countries were focusing on their domestic concerns, India showed exemplary management of COVID-19 not only at the domestic level rather pursuing its health diplomacy towards many regions like Asian, African, and Latin American and Caribbean countries [49,50]. Rajani Mol et al., in a recent humanitarian and geopolitical analysis in the context of Indo-African relations, highlighted that through GHD and vaccine diplomacy, India has become a global healthcare provider in the African continent and has achieved impressive gains through its soft power diplomacy by becoming a compassionate and benevolent actor in the African continent [51].

The African continent has been figuring very prominently in India’s health diplomacy. As a result of proactive health and medical diplomacy, India had supplied medicines such as hydroxychloroquine, paracetamol, several other drugs, and vaccines to about 25 African countries. India has also provided medical kits telemedicine support partnered with top national institutions such as All India Institute of Medical Sciences, Raipur and other local institutions. India has been providing medical training to the frontline health workers of many African countries to combat and control the pandemic crisis [52]. At the pandemic’s peak, India re-emphasized the Africa Focus Program, launched in April 2002. In this program, the Indian Foreign Affairs had conversations with several African countries like Burkina Faso, Mali, Niger, Nigeria, Uganda, etc., and promised medical assistance and support [53]. Subsequently, medical assistance was provided to another twenty African countries. India has provided vaccines to many African countries such as Morocco—-7,000,000; Mauritius—200,000; Seychelles—5000; Egypt—50,000; Algeria—50,000; South Africa—1,000,000; Ghana—600,000; Congo—1,716,000; Angola—624,000; Nigeria; Kenya—1,020,000; Lesotho—36,000; Rwanda—200,000; Democratic Republic of Senegal—324,000 etc. [54].

Being a ‘world’s pharmacy’, India had contributed significantly to meet the global demand for vaccines and low-cost generic drugs. About 20% of pharmaceutical exports of value of US dollar 70 billion have been exported to the African continent in general, and southern and western African countries are the largest beneficiaries of Indian medicines. This list also includes antiretroviral (ARV) drugs. The ARV drugs are very cheaper compared to the Western companies’ products. India has also played a significant role in capacity building and collaboration in the health sector. Moreover, the major Indian healthcare providers and pharmaceutical companies have collaborated with their African partners in many ways, such as opening super-specialty hospitals across Africa. India has also been offering telemedicine services to African countries more proactively. However, the pan-African network is supported by India. It has been offering telemedicine services since 2009 to African countries. The sources of telemedicine networks revolutionized the medical healthcare system of the continent as a strong link has been established between the Indian hospitals and educational centers with their African counterparts [55].

The third relates to India’s active “medical diplomacy.” Africa’s reliance on a cheap supply of essential medicines in addition to an affordable COVID-19 vaccine is only likely to increase in the near future. Yet Africa’s success in containing pandemics such as Ebola offers lessons to India, too. An illustrative example is Senegal, which has adapted its experiences from the 2014 Ebola outbreak to fight COVID-19. Furthermore, numerous additional lessons on disease control from African countries can also be scaled to improve India’s health sector. India appears well-poised to share its digital capabilities for improved and affordable access to universal healthcare. New Delhi has revamped its telemedicine and online video consultation infrastructure on the continent. This offers a cost-effective and safe option for treating contagious diseases. A major challenge is improving access to generic medicines through negotiated intellectual property rights waivers. At the WTO, India and South Africa have taken a moral stance against “vaccine nationalism”. Nevertheless, the organization rejected their joint proposal in October 2020 for a temporary waiver on drugs and COVID-19 vaccines. It would be worth quoting Dr. Piyush Gupta, associate director, GNH India, who said, “Regulatory harmonization in the African continent is a welcome step. It will positively impact India’s export in Africa to strengthen India’s regulatory compliance in African countries. It will do away with the need to register certain life-saving drugs in every member state of AU. Once the product gets regulatory approval in a member state, it can be distributed to other states. It will further boost India’s export in Africa” [56].

### 4.6. Impact of China’s Health Diplomacy

The African continent has been figuring very prominently in Chinese foreign policy. During the last couple of years, the multilateral cooperation between China and the African countries has grown exponentially. China has launched several policy-free works and programs to carry these relations to the pinnacle. The adversity of endemic COVID-19 has provided an opportunity to cement these relations. On the part of China, health diplomacy has been embraced as one of the strategies to further promote these relations. Since the pandemic, China has been pursuing its multipronged globalized health diplomacy in general and towards the African continent in particular [45]. Chinese health diplomacy includes many actors such as the state, the provincial governments, multinational companies, and entrepreneurs.

The Chinese health diplomacy towards Africa has included healthcare facilities, capacity building, training facilities, etc. [57]. The African continent has been made one of the important regions for Chinese health diplomacy. China sent the first medical team to Algeria during the former premier Zhou Enlai in 1963 [58]. Since then, China has been consistently pursuing its health diplomacy towards Africa. The Chinese health diplomacy towards Africa includes construction of healthcare facilities, provision of medical aid, medical doctors, and training the African medical staff. At the beginning of the 21st century, China launched its Africa policy (2006). Under this policy, the Chinese government formulated its priority, particularly in terms of health-related issues. China has committed to promoting effective treatment services for diseases such as malaria by sending medical teams, providing medical equipment, and training doctors throughout the African continent. The policy also committed research assistance for traditional medicine for the management of HIV/AIDS [59]. Moreover, during the African Cooperation Forum (CACF) in 2006, the Chinese government promised to provide 5 billion dollars in preferential loans to the African continent. It underlined the importance of health and education in African aid efforts. As per the output of the Chinese health ministry, about 1700 medical workers had been sent to the 14 African countries by the end of 2010.

In the context of the COVID-19, China has not only successfully combated and limited the epidemic but has also capitalized on the emerging chance to refocus on its health diplomacy. Apart from the Chinese governments, the private players have also been part of Chinese health diplomacy. In this part, Alibaba’s Jack Ma and the Alibaba foundation have contributed immensely to the African countries to combat the pandemic. The same has provided several thousands of detection kits, PPE face masks, infrared thermometers, surgical masks, hand gloves, and extraction kits. Several Chinese companies have played an active role in the region by donating to local charity institutions, non-governmental organizations, and civil societies. However, the major expectation from China on the part of the African countries is that apart from sharing medical knowledge and medical supplies, the Chinese government must waive the loans and arrangements of easy repayments. Chinese health diplomacy has played a spectacular role in cementing the ties between China and the African countries. Diplomacy has created the benevolent image of China in the minds of political leaders and the common people of the African countries. This argument can be substantiated by the fact that the Pew Global Attitudes Project Survey showed that most people in African countries held China in high esteem and perception of considerable control over African countries.

In terms of Chinese diplomacy, vaccines, medications, and healthcare facilities, African countries’ experiences have not been disappointing. It is perceptibly clear from the argument of Njenga, Chair of the NCDs Alliance of Kenya [60], that Africa’s inadequate regulatory systems had made the continent more vulnerable to the possibility of sub-standard, ineffective, or defective vaccines and medicines even by way of “gifts.” In the same way, it has been further argued that it is difficult to share and transfer the vaccine know-how with big pharma industries based in rich countries in the West who are eager to sell to the highest bidders leaving the lower-middle-income countries (LMICs). Therefore, the vaccines entering the continent are either “unregulated, or to a lesser extent, what doesn’t meet regulatory standards in the West is often offered or passed on to the African continent,” as per the statement by Dr. Njenga. Since pharmaceuticals are among the most traded goods in Africa, the Free Trade Agreement, in the absence of an AMA, could potentially widen the scope of poor-quality, poorly regulated pharmaceuticals being traded across multiple borders with little or no oversight or recourse, to the detriment of all Africans.

### 4.7. Future of Africa with African Medicines Agency in Action

In general, all vaccines need mandatory approval from their national regulatory authorities, and according to a South African study by Ncube et al., across Africa, these regulatory authorities range in quality from “robust and functional” to offering regulation that is “virtually non-existent” [61]. For decades, Africa has imported 99% of its vaccines. The COVID-19 pandemic has prompted to accelerate the efforts to establish AMA similar to EMA, which would provide national regulators in the African region with regulatory guidance on new medicines and vaccines [62]. As highlighted by Ncube et al., the AU Model Law and the AMA hold the promise of addressing inadequacies and discrepancies in national regulatory legislation and ensuring effective medicines regulation at the continental level by galvanizing technical support regulatory expertise, and resources [61]. The goal of the AU Model Law is to encourage cross-national collaboration and ensure that promising medical innovations are developed, evaluated, and scaled up for greater health impact [63,64,65,66,67]. The AU Model Law also supports the AU’s purpose of promoting local pharmaceutical manufacture for public health and economic progress [4] and continental initiatives to advocate for and catalyze access to new medical products for people in need [12,18,65]. Moreover, Chattu et al. emphasized that the AU should leverage the momentum of the rise of GHD to navigate the politics of global health governance in an interconnected world and develop robust preparedness and disease response strategies. They further argue that the AU, RECs, and African Centers for Disease Control should harmonize their plans and strategies by aligning them towards a common goal that integrates health in African development agendas [3]. Thus, the need for AMA becomes a top priority for addressing the huge gaps by strengthening regulations, improving access and boosting manufacturing [68]. The African Union Commission, its agencies, and the Member States need to support AMA’s strengthening for several reasons. AMA is an ideal target for such a concerted effort in the context of an African Agenda 2063, where socio-economic growth is driven by strong, well-governed national, sub-regional, and continental institutions. There has never been a more pressing need for Africa to have its own regulatory body for medicines and medical products than during the current COVID-19 era, which has exposed the limitation of dependence on other nations’ goodwill in LMICs are faced with an existential challenge. In this context, the AMA is expected to play a stronger role and become the default regulator for African countries, which are still far behind in creating their national capacity in the pharmaceutical sector’s regulatory affairs.

Creating a strong AMA should not be perceived as a zero-sum game that erodes the advantages of other bodies either. Rather, a strong AMA, working in close partnerships created through global health diplomacy, would be the ideal partner for European and other regional regulatory authorities seeking to stem the tide of counterfeit, sub-standard, or fake products. For example, the recent reports on substandard, falsified, or diverted vaccines available in the Medicine Quality Monitoring Globe from 12 March 2020 to 5 March 2021 extracted from Medical Product Quality Report- COVID-19 vaccine issues are shown in Appendix A and Appendix B Moreover, expansion of pharmaceutical manufacturing capacity in Africa under licenses from European and North American companies will find in the AMA a reliable partner that helps maintain the manufacturing process and product standards for their new investments in Africa. The COVID-19 pandemic has demonstrated how global health security demands will often be challenged by limited production capacity in the global north, from testing kits to personal protection equipment to key medicines to vaccines. Even the strong efforts by G20 countries such as India might often fail to satisfy the urgent commodity production needs during such global crises.

Now is an opportune time to advance and strengthen the AMA. The newly elected Nigerian leader of the World Trade Organization has joined the African Union Commission’s call for expanding the manufacturing (and, by extension, the regulatory) capacity for COVID-19 commodities in LMICs. Non-African manufacturers of COVID-19 vaccines have begun to suffer manufacturing setbacks, constrained capacity, and political pressure from the authorities of the countries where they are based. The scene is set for accelerating an AMA that can oversee and regulate several African countries’ ability to manufacture such commodities in good quality, quantity, and speed. The recent proposal to waive off certain provisions of the TRIPS for the prevention, containment, and treatment of COVID-19 by India and South Africa at the WTO presents an important opportunity for all African governments to unite and stand up for public health, global solidarity, and equitable access and a functional AMA would serve a great purpose for similar challenges in the future [69]. As highlighted by Taghizade et al., GHD proves to be very useful for negotiating better policies, stronger partnerships and achieving international cooperation in this phase with many geopolitical shifts and nationalist mindset among many nations at this stage of COVID-19 vaccine roll-out [70]. Moreover, Binagwaho et al. have highlighted that the pharmaceutical companies should share their technologies to increase the supply and reduce the prices; the governments should prioritize equitable distribution to the most at- risk in LMICs and should bolster their logistical capacity in preparation for mass vaccination campaigns [71]. In this context there is another great example of South Africa’s GHD by Modisenyane and colleagues have highlighted that in collaboration with the global advocacy movement, catalyzed the mobilization of support for universal access to ARV treatment on a national and global scale, as well as the promotion of healthcare access as a human right [72]. Therefore, in this context, the African countries should get the AMA in action and have a proactive role in handling the anticipated challenges of the post-COVID-19 era.

## 5. Conclusions

Globally, access to medicines is increasingly a multi-dimensional and multi-industrial public health issue. The AHS 2016–2030 emphasized regulation of medical products and technologies at the continental level for the availability of quality products addressing the equity issues, as well. AHS also stated that UHC must be supported with an adequate supply of commodities, including essential medicines, contraceptives, condoms, vaccines, effective drugs, and other supplies. Therefore, AMA was founded as a key tool for enhancing regulatory oversight of medicines and vaccines across 54 countries. Since the AMA Treaty is still to be ratified, the role of GHD is very critical to influence the member countries to quickly enact the relevant laws in their national parliaments and bring the new governance structure of AMA into existence. AMA is expected to sow the seeds of regulatory excellence and sustainability. However, it is important to note that the world is going through an unprecedented pandemic that has affected almost all aspects of human lives. A functional AMA created through GHD can play a significant role in utilizing TRIPS flexibilities extension and can be an ideal partner for other regional and global stakeholders such as regulatory authorities, funding agencies, multilaterals, and national governments to halt counterfeit, sub-standard, or fake products. AMA will coordinate prevention, detection, and response to counterfeit and falsified medicines. Therefore, considering the activities of AMA, it will only be beneficial to enhance collaboration, cooperation, and mutual growth by ratification of the AMA Treaty, which will be a big game-changer for the pan-African region in this post-pandemic world. Further, AMA with strong regulatory expertise and harmonized legal frameworks enhance successfully adopting TRIPS flexibilities to ensure equitable distribution in the COVID-19 vaccine roll-out.

## Figures and Tables

**Table 1:** Main Functions of African Medicines Agency as per the Treaty.

S. No	Broader Function	Specific Activities
1	Coordinate (5)	ongoing initiatives to harmonize medical products regulation; the collection, management, storage, and sharing of information on all medical products, including SF medical products, with all its States Parties and globally; joint reviews of applications for the conducting of clinical trials and provide technical support in quality control of drugs at the request of Member States; and collaborate the inspection of drug manufacturing sites, including the regulatory oversight and safety monitoring of medical products, as determined by State Parties and/or the AMA; access to and network the services available in quality control laboratory services within national and regional regulatory authorities.
2	Convene (1)	6. in collaboration with the WHO, the AMRC, and other bodies, meetings related to medical products regulation in Africa.
3	Designate (1)	7. promote, strengthen, coordinate, and monitor RCOREs to develop the capacity of medical products regulatory professionals.
4	Develop (1)	8. systems to monitor, evaluate, and assess the comprehensiveness of national medical products regulatory systems with the view to recommending measures that will improve efficiency and effectiveness.
5	Evaluate (1)	9. and decide on selected medical products, including complex molecules, for treatment of priority diseases/conditions as determined by the African Union and WHO.
6	Examine (1)	10. discuss and/or express regulatory guidance on any regulatory matter within its mandate, either on its initiative or at the request of the African Union, RECs, or State Parties.
7	Monitor (1)	11. the medicines market through the collection of samples in every State Party to ensure the quality of selected drugs, have them analyzed and provide the results to States Parties and other interested parties, who will thus have reliable information on the quality of the drugs circulating in their countries and, where necessary, will take appropriate measures.
8	Promote (3)	12. the adoption and harmonization of medical product regulatory policies and standards and scientific guidelines and coordinate existing regulatory harmonization efforts in the RECs and RHOs; 13. and advocate for adopting the AU Model Law on medical products regulation in States Parties and RECs to facilitate regulatory and legal reforms at continental, regional, and national levels; 14. cooperation, partnership, and recognition of regulatory decisions in support of regional structures and NMRAs that considers mobilization of financial and technical resources to ensure the sustainability of the AMA.
9	Provide (4)	15. guidance on the regulation of traditional medical products; 16. advice on the marketing authorization application process for the priority drugs described by the States Parties or on the products proposed by the pharmaceutical laboratories; 17. regulatory guidance, scientific opinions, and a common framework for regulatory actions on medical products, as well as priority and emerging issues and pandemics in the event of a public health emergency on the continent with cross border or regional implications where new medical products are to be deployed for investigation and clinical trials; 18. technical assistance and resources, where possible, on regulatory matters to States Parties that seek assistance and pool expertise and capacities to strengthen networking for optimal use of the limited resources.

Source: Article 6 of the Treaty of the AMA.

## Data Availability

The data presented in this study are available on request from the corresponding author.

## Appendix A

Reports on substandard, falsified or diverted vaccines available in the Medicine Quality Monitoring Globe from 12 March 2020 to 31 December 2020. (Medical Product Quality Report-COVID-19 vaccine issues).

## Appendix B

Reports on substandard, falsified or diverted vaccines available in the Medicine Quality Monitoring Globe from 5 January 2021 to 5t March 2021. (Medical Product Quality Report-COVID-19 vaccine issues).
